# Antimicrobial Photodynamic Therapy Combined With Antibiotic in the Treatment of Rats With Third-Degree Burns

**DOI:** 10.3389/fmicb.2021.622410

**Published:** 2021-02-24

**Authors:** Zhanjuan Zhao, Jinduo Ma, Yiyi Wang, Zehua Xu, Lu Zhao, Jianxi Zhao, Ge Hong, Tianjun Liu

**Affiliations:** ^1^College of Basic Medicine, Hebei University, Baoding, China; ^2^Tianjin Key Laboratory of Biomedical Material, Institute of Biomedical Engineering, Chinese Academy of Medical Sciences and Peking Union Medical College, Tianjin, China; ^3^College of Clinical Medicine, Hebei University, Baoding, China; ^4^College of Bioscience and Resources Environment, Beijing University of Agriculture, Beijing, China; ^5^College of Public Health, Hebei University, Baoding, China; ^6^Medical Oncology, Affiliated Hospital of Hebei University, Baoding, China; ^7^Department of Radiology, Affiliated Hospital of Hebei University, Baoding, China

**Keywords:** antimicrobial effect, antimicrobial photodynamic therapy, photosensitizer, cationic porphyrins, burned, rats

## Abstract

Cationic porphyrin conjugate, protoporphyrin IX-methyl ethylenediamine derivative (PPIX-MED) has a potent photosensitive antibacterial effect on clinically isolated bacteria, including methicillin-resistant *Staphylococcus aureus*, (MRSA), *Escherichia coli*, and *Pseudomonas aeruginosa*. This study investigated (i) the PPIX-MED-mediated antimicrobial photodynamic effect on these three species *in vitro* and (ii) the effect of antimicrobial photodynamic therapy (aPDT) combined with the use of an antibiotic on the healing *in vivo* of third-degree burns of rats with the wounds infected by these bacterial species. PPIX-MED exerted a potent inhibitory effect on the growth of the three bacterial species by producing reactive oxygen species when photoactivated. PPIX-MED-mediated antimicrobial photodynamic therapy (PPIX-MED-aPDT) had high bacterial photoinactivation ability *in vitro*, with a minimum inhibitory concentration of 15.6 μM PPIX-MED against each of the three types of bacteria and minimum bactericidal concentrations of 31.25 μM against MRSA and *E. coli* and 62.5 μM against *P. aeruginosa.* In rats with third-degree burns infected by a mixture of these bacteria, the bactericidal efficiency of PPIX-MED–aPDT-combined-with-antibiotic treatment was higher than that of antibiotic or aPDT treatment alone. This was confirmed by analysis of viable bacterial counts in wound tissue and blood. Enzyme-linked immunosorbent assay revealed that aPDT-combined-with-antibiotic treatment resulted in an obvious reduction in tumor necrosis factor-alpha and interleukin-6 levels compared with the no-treatment control group and the other treatment groups. Immunohistochemistry revealed that the expression of basic fibroblast growth factor and CD31 (a marker of neovascularization), expressed in burn wound tissue was higher in the aPDT-combined-with-antibiotic treatment group than in the other groups. PPIX-MED–aPDT has a promising bactericidal effect both *in vitro* and *in vivo*, and PPIX-MED–aPDT-combined-with-antibiotic treatment enhanced the healing of infected third-degree burns in rats.

## Introduction

Antimicrobial drugs are overused, misused, and widely applied prophylactically, which results in the emergence of drug-resistant microorganisms (Park et al., 2012). Multidrug-resistant bacterial infections are a major challenge to healthcare and an important cause of morbidity and mortality in hospitalized patients (Hamblin et al., 2002; Oda et al., 2004; Davies and Davies, 2010; Jernigan et al., 2020). The skin is the first line of defense against invading pathogens; it acts as a physical barrier against microbial invasion. Burning causes a rupture in the skin and other epithelial layers, which exposes the individual to infection as open wounds allow bacteria to enter and are suitable environments for their survival (Oyama et al., 2020). Infection is a common problem in cutaneous wounds and a critical complication in wound healing (Oyama et al., 2020). All wounds will have some bacterial colonization. Methicillin-resistant *Staphylococcus aureus* (MRSA), *Escherichia coli*, and *Pseudomonas aeruginosa* are early colonizers, accounting for the majority of burn wound infections (Cetik Yildiz et al., 2019). Burn healing is a complex process that involves clotting, inflammation, granulation tissue formation, epithelialization, collagen synthesis, and tissue remodeling (de Vasconcelos Catão et al., 2015). A delay or failure in the treatment of wounds can lead to progressive bacterial colonization until the development of a systemic infection (Oyama et al., 2020). As such, anti-infection treatment is important for burn patients (Mai et al., 2017).

Antibiotic treatment has side effects, which are themselves a threat to the health of burn patients. For deep burn wounds, antibiotic use may hinder the regeneration of skin tissue and the treatment of edema. Therefore, the development of novel antibacterial therapies, especially for burn infections caused by multidrug-resistant bacteria, is urgently required. One treatment shown in the literature to be lethal to microbial pathogens and to accelerate the healing of burns is antimicrobial photodynamic therapy (aPDT) (Seegers et al., 2003; Mai et al., 2017). aPDT involves three components: oxygen, a photosensitizer, and laser light with a wavelength matching the absorption of the photosensitizer. aPDT is based upon energy transfer from light to oxygen to produce reactive oxygen species that are lethal to microbial pathogens (Bhatti et al., 1998; Tavares et al., 2010). The antibacterial activity of aPDT results from oxidative damage produced by singlet oxygen and other reactive species; bacteria will not be able to develop resistance to it (Hamblin et al., 2003; Luan et al., 2009; Dai et al., 2010; Park et al., 2012).

The photosensitizer is the crucial element in aPDT. Cationic photosensitizers are capable of efficiently killing Gram-negative bacteria, inhibiting the secretion of inflammatory factors, and promoting wound healing after infection of burns (Mai et al., 2017; Huang et al., 2018). We hypothesized that the protoporphyrin IX (PPIX) conjugation, which is cationic, can exhibit significant phototoxic activities against Gram-negative and Gram-positive bacteria. A few reports have been published on the effect of aPDT on burn healing (Garcia et al., 2010; de Vasconcelos Catão et al., 2015; Jernigan et al., 2020). Rat burn wounds infected with bacteria can progress to invasive and life-threatening infections, such as bacteremia, abscesses, pneumonia, and sepsis (Mai et al., 2017). In the treatment of burn infections, antibiotic medication should be administered throughout the body instead of local administration, which leads to a low concentration of antibiotics at the burn site, making it impossible to kill the bacteria completely. Therefore, aPDT, a beneficial supplement to antibiotic treatment, can directly act on the infected lesions to effectively achieve antibacterial and cleansing results. At the same time, the low-level laser irradiation can also promote wound healing (de Vasconcelos Catão et al., 2015). aPDT can be used externally, but the body needs its immune system, possibly also antibiotics, to resist bacteria internally. Using aPDT to remove surface bacteria and antibiotics to inhibit bacteria in the body, a small quantity of antibiotic can have a therapeutic effect.

**FIGURE 1:**
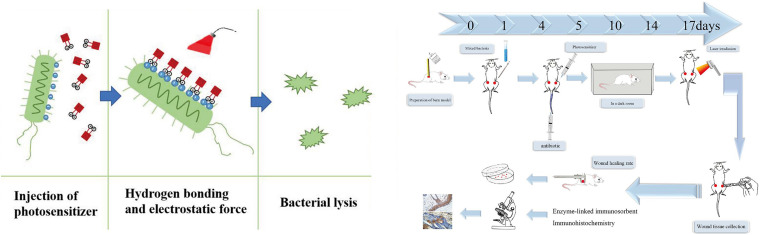
Diagrammatic representation of the model of skin burn wound infection treatment by antimicrobial photodynamic therapy.

The principal aim of this study was to evaluate the effectiveness of aPDT *in vivo* on healing of experimental rat burn wounds infected with a mixture of pathogenic bacteria. The wounds were treated by aPDT using the photosensitizer PPIX-MED (Figure 1) with and without additional intravenous antibiotic treatment (Hamblin et al., 2002; Oda et al., 2004; Davies and Davies, 2010; Jernigan et al., 2020).

## Materials and Methods

### Chemicals and Instruments

**FIGURE 2:**
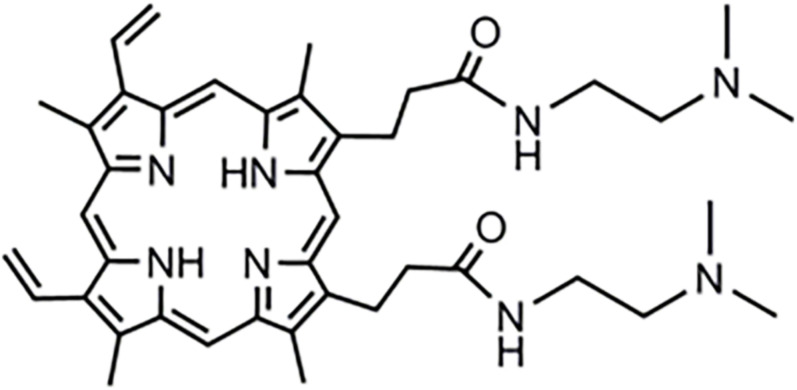
Structure of (C_42_H_54_N_8_O_2_) dimethyl-8,13-divinyl-3,7,12,17- tetramethyl-21H, 23H-porphyrin-2,18-*bis*[-*N*-2-(dimethylamine)ethyl] propenamide (PPIX-MED).

The compound PPIX-MED was synthesized in the Key Laboratory of Biomedical Material, Institute of Biomedical Engineering, Peking Union Medical College, and Chinese Academy of Medical Sciences. The chemical structure of this porphyrin derivative is shown in Figure 2. A stock solution (500 μM) was prepared by dissolution in dimethylsulfoxide (DMSO) and was stored at −20°C in the dark before use.

Ceftriaxone sodium is a broad-spectrum antibiotic, which was provided by Harbin Pharmaceutical Group (Harbin, China).

### Light Source

A semiconductor 650-nm laser (7404, Industry, United States) was selected for this study, and the light spot energy density was determined with an optical power meter (LM1; Carl Zeiss, Germany).

### Photobleaching

Photobleaching was conducted with a Multimode Microplate Spectrophotometer (Lv et al., 2013). Briefly, dye and buffer solutions were prepared immediately before measurements. Samples (200 μl) of 2 × 10^–5^ M dye in 96-well microtiter plates were sealed with cover slips to avoid evaporation. Photobleaching measurements at 300–800 nm were conducted for 30 min at ambient temperature, and data were recorded every 5 min. The delivered light energy was 0.2 J/cm^2^ per min, and the total light energy density was 6 J/cm^2^.

### Measurement of the Quantum Yield of Singlet Oxygen

Singlet oxygen quantum yield ([Φ_Δ_]) experiments were performed according to Álvarez-Micó et al. (2007). Briefly, [Φ_Δ_] was determined by the decomposition of 1,3-diphenyl isobenzofuran (DPBF) in DMSO; [Φ_Δ_] correlated with the decay of the absorption of DPBF at 410 nm (Lau et al., 2011). The sample was irradiated at 650 nm, 6 J/cm^2^. Equation 1 was used to calculate the singlet oxygen generation, with 5,10,15,20-tetraphenylporphyrin (TPP) used as the reference ($\left[\Phi_{\Delta }^{\text{R}}\right]$ = 0.64) (Álvarez-Micó et al., 2007).

(1) $$\left[\Phi_{\Delta }^{S}=\Phi_{\Delta }^{R}\frac{{\text{K}}^{\text{S}}{\text{F}}^{\text{R}}}{{\text{K}}^{\text{R}}{\text{F}}^{\text{S}}}\right]$$

where $\left[\Phi_{\Delta }^{R}\right]$ is the singlet oxygen quantum yield for the reference, superscripts S and R indicate the sample and reference compound, respectively, *K* is the slope of the plot of change difference in absorbance of DPBF (at 410 nm) with irradiation time, and *F* is the absorption correction factor, which is given by *F* = 1 −10^–OD^, where OD is the absorbance at the irradiation wavelength.

### Bacterial Culture

Three clinical bacterial strains – MRSA, *E. coli*, and *P. aeruginosa* – were isolated at the Tianjin Armed Police Hospital. Luria–Bertani (LB) medium was used to culture these bacterial strains. A single colony was used to inoculate 10 ml of liquid medium. The cells were grown at 37°C in aerobic conditions in a shaking incubator (200 rpm) until an optical density at 600 nm (OD_600_) of approximately 0.7 was reached. The cells were then harvested by centrifugation and resuspended in an equal volume of phosphate-buffered saline (PBS) (Zhao et al., 2014).

### Fibroblast Culture

Mouse fibroblast NIH 3T3 cells (SCSP-515) (Jainchill et al., 1969; Gong et al., 2018) were purchased from the Cell Bank of the Chinese Academy of Sciences (Shanghai, China). The cells were cultured in Dulbecco’s modified Eagle’s medium (Sigma-Aldrich, United Kingdom) containing 10% heat-inactivated fetal bovine serum (Gibco), penicillin (100 U/ml), and streptomycin (100 μg/ml) (Sigma-Aldrich) and incubated at 37°C in a humidified atmosphere of 5% CO_2_ until the cell monolayer reached at least 80% confluence. The cells were washed with PBS and incubated for 3 min at 37°C with 0.05% trypsin and 0.02% ethylenediaminetetraacetic acid, seeded into 96-well cell culture plates (8 × 10^3^–1 × 10^4^ cells per well), and incubated overnight. All subsequent photoinactivation experiments involving these cells were performed in 96-well cell culture plates.

### Cell Survival Assay

After 24 h of cell growth at 37°C in 5% CO_2_, the cells were incubated with PPIX-MED at various concentrations (0, 3.9, 7.8, 15.6, and 31.25 μM) at 37°C for 30 min in the dark, then irradiated with 6 J/cm^2^ light, and incubated overnight at 37°C. At 24 h after antimicrobial photodynamic therapy, 5 mg/ml 3-(4,5-dimethylthiazol-2-yl)2,5-diphenyl tetrazolium bromide (MTT) (Thermo Fisher, United States) was added to the cells, which were incubated at 37°C to allow cleavage of the tetrazolium ring by mitochondrial dehydrogenases and the formation of blue formazan crystals in living cells. After 3 h, the supernatant was removed, and the crystals were dissolved in DMSO. The absorption of formazan in each well was determined at 490 nm using a microplate reader (Thermo, Varioskan Flash Multimode Reader). All assays were performed in the dark (Kashef et al., 2012; Ahmed Alamoudi et al., 2018).

### Dose-Dependent Photoinactivation Effects

The number of bacterial colonies is described in terms of the number of colony-forming units (CFU). Mixtures of 1 × 10^7^ CFU/ml bacterial suspension and different concentrations of PPIX-MED (0, 3.9, 7.8, 15.6, and 31.25 μM) were added to a 96-well plate, incubated in the dark at 37°C for 30 min, and then irradiated with laser at 6 J/cm^2^. After that, 100 μl of the mixture was taken from each well, and a gradient dilution (1 × 10^–1^, 1 × 10^–2^, 1 × 10^–3^, 1 × 10^–4^, and 1 × 10^–5^) was spread on LB agar plates, which were incubated at 37°C for 18 h in the dark. Then, the number of CFU was counted. The experiment was repeated three times. Bacterial survival fractions were expressed as the ratio of the number of CFU of bacteria treated with light and photosensitizer to the number of CFU of untreated bacteria (Grinholc et al., 2008).

### Uptake Assay

Uptake experiments were performed according to the method of Soukos et al. (1998). In general, 1 ml bacterial suspension was centrifuged (9000 × *g*, 1 min), and the cells were resuspended in PBS to OD_600_ = 0.7. PPIX-MED was added to final concentrations of 3.91–62.5 μM. The mixture was incubated in the dark for 30 min at ambient temperature and centrifuged at 9000 × *g* for 1 min; then, the cells were washed with PBS to remove residual photosensitizer. The bacterial pellet was dissolved in 1 ml 10% aqueous sodium lauryl sulfate solution and left for 24 h to fully release the absorbed photosensitizer. The uptake of photosensitizer by the bacteria was determined by a fluorescence assay. The PPIX-MED fluorescence was read (*λ*_ex_ = 406 nm, *λ*_em_ = 604 nm) and normalized as described above. A standard curve was obtained by plotting known concentrations of the target compound against the fluorescence intensity. The uptake amount was calculated by comparing the determined fluorescence intensity with the standard curve. A blank control group without photosensitizer was also used.

### Determination of Minimum Inhibitory Concentration and Minimum Bactericidal Concentration

The three bacterial strains used in this study were treated with the same procedure; as an example, the treatment of MRSA is described in detail. Experiments were performed in 96-well flat-bottomed plates. Twenty microliters of MRSA suspension and 180 μl of the PPIX-MED compounds were added to each well. The final concentration of bacteria in the mixture was 10^6^ CFU/ml. PPIX-MED was prepared at 1.95, 3.9, 7.8, 15.6, 31.25, 62.5, 125, 250, and 500 μM. The plates were kept in the dark for 30 min at 37°C and then exposed to light for 30 min or kept in the dark to provide dark control samples. Then, the samples were incubated in the dark at 37°C, and the number of CFU was evaluated after 18 h. Three sets of independent experiments were performed (Rodrigues et al., 2013).

### Fractional Inhibitory Concentration Index

To determine the fractional inhibitory concentration index (FICI), ultrapure water was used to dissolve the ceftriaxone sodium and PPIX-MED so as to give stock concentrations of 4, 2, 0.5, and 0.25 MIC. Using the checkerboard design method, 50 μl of each drug was added at different concentrations in the horizontal and vertical columns of a 96-well plate, respectively. Then, 100 μl of bacterial (10^6^ CFU/ml) suspension was added and mixed by slight shaking. The plates were kept in the dark for 30 min at 37°C and then exposed to light for 30 min. After light exposure, the samples were incubated in the dark at 37°C for 16–20 h. The MIC of each antimicrobial in the combination was read and interpreted using previously described methods. For each antimicrobial combination, we calculated the FICI by computing the ratio of the MIC of the combination divided by the MIC of the antimicrobial alone for each agent and then adding those two ratios together (see Equation 2). The FICI data were interpreted using the following criteria: synergy, FICI ≤ 0.5; indifference, FICI 0.5–4.0; and antagonism, FICI > 4.0 (Barbee et al., 2014).

(2) $$\text{FICI}=\left[\frac{{\text{MIC}}_{A\left(\mathrm{with}B\right)}}{{\text{MIC}}_{A\left(\mathrm{alone}\right)}}\right]+\left[\frac{{\text{MIC}}_{B\left(\mathrm{with}A\right)}}{{\text{MIC}}_{B\left(\mathrm{alone}\right)}}\right]$$

### Imaging by Confocal Laser Scanning Microscopy

The bacterial strains were suspended in PBS to an appropriate cell density (OD_600_ = 0.7), and then they were treated with 25 μM PPIX-MED for 30 min at room temperature. The cells were then harvested by centrifugation (9000 × *g*, 1 min), washed twice with PBS, and resuspended. One drop of this suspension was placed onto a confocal dish and allowed to dry. Fluorescent images were taken with a confocal laser scanning microscope (LSM510; Carl Zeiss), with excitation at 405 nm and emission at 650 nm (Zhao et al., 2014).

### PPIX-MED–aPDT *in vivo*

#### Third-Degree Burn Wound Model and Establishment of Infection

The effects of PPIX-MED–aPDT on burn wound healing were evaluated using a rat model. All animal experiment procedures were experimented according to the National Institutes of Health Guide for Care and Use of Laboratory Animals, and the protocol was approved by the Laboratory Animal Management Committee/Laboratory Animal Welfare Ethics Committee, Institute of Radiation Medicine, Chinese Academy of Medical Sciences.

Sprague–Dawley rats from Beijing HFK Bioscience Co., Ltd., weighing about 220 g, were housed at one rat per cage and maintained in the dark except during aPDT treatment. The rats were first anesthetized via intraperitoneal injection of 1% sodium pentobarbital (80 mg/kg). Then, their back was shaved with an electric razor, followed by the use of a depilatory agent. In sterile conditions, a hollow round tube with a diameter of 30 mm was tightly attached to the back of the rat. Then, 50 ml of boiling water was poured into the empty pipe for 20 s. On each side, a burn wound was made; the left and right sides were symmetrical. After the scalding, an abdominal injection of normal saline (40 ml/kg) was used to resist shock. Immediately after that, a suspension (50 μl) of mixed bacteria (10^9^ CFU/ml MRSA, 5 × 10^8^ CFU/ml *E. coli*, and 5 × 10^8^ CFU/ml *P. aeruginosa*) in sterile PBS was inoculated onto the surface of each wound with a pipette tip and then smeared onto the wound surface with an inoculating loop. Then, the rats were used as wound model for mixed bacterial infection.

### Photodynamic Treatment Protocol

Forty rats with burn wounds infected by mixed bacteria as described above were randomly divided into four groups: (A) no treatment (control group), (B) aPDT group (100 μl PPIX-MED each wound + laser treatment), (C) antibiotic group (intravenous injection of ceftriaxone 0.8 ml; ceftriaxone sodium was diluted to a concentration of 200 mg/kg in ultrapure water medium immediately before use), and (D) aPDT + antibiotic group (100 μl PPIX-MED each wound + laser therapy + intravenous injection of antibiotic 0.8 ml mixed treatment).

At 24 h after infection, 100 μl of PPIX-MED solution [four times of minimum bactericidal concentration (MBC) against *P. aeruginosa*] was injected under the eschar of the wound of rats in groups B and D. Then, after 30 min, these rats were illuminated with a 650-nm laser for 10 min (total light energy density 60 J/cm^2^). On the next day, the same light dose was given to increase the effect of PPIX-MED–aPDT, but no more PPIX-MED was administered. This treatment is defined as one treatment. The above-mentioned treatment was repeated four times (total treatment days = 8). In groups C and D, the rats underwent a tail intravenous injection of ceftriaxone 0.8 ml once every 2 days; the above-mentioned treatment was repeated four times.

#### Bacterial Loads

To determine the bacterial counts in tissue samples at 4, 10, and 14 days post-infection, 10% tissue homogenates was serially diluted in PBS (1:10, 1:100, 1:1,000, 1:10,000, 1:100,000, and 1:1,000,000) and plated on a common broth–agar plate in triplicate. The plates were then incubated for at least 18 h at 37°C in a humidified atmosphere. Colony counts were expressed as log_10_ CFU per gram of tissue or milliliter of wound fluid.

#### The Rate of Wound Healing

After the treatment, wound healing was observed, and the width and length of the wounds were measured using a vernier caliper on days 5, 10, 14, and 17 post-infection. The rate of wound healing of each group after infection was recorded.

#### Blood Culture

After 4, 10, and 14 days post-infection, 1 ml of blood was extracted from the femoral vein of rats in sterile conditions, then placed in a test tube containing 9 ml of liquid LB medium, mixed thoroughly, and placed in an incubator at 37°C. After 3 days, 100 μl of ordinary coating inoculated on agar plates was taken and incubated for 24 h. If there was bacterial growth, the blood culture was considered positive.

#### Enzyme-Linked Immunosorbent Assay

ELISA was performed as described by Zhang et al. (2015). Resected skin tissue samples were frozen in liquid nitrogen, pulverized or homogenized, and digested in a tissue lysis reagent. Tumor necrosis factor (TNF-α) and interleukin-6 (IL-6) production at the skin injury site was quantified using ELISAs in accordance with the manufacturer’s protocols (R&D Systems, Minneapolis, MN, United States).

#### Immunohistochemistry of Wound Tissue

After 4, 10, and 17 days post-infection, the skin granulation tissue at the forefront of the new epithelium was removed from the wound in aseptic conditions. Immunohistochemistry (IHC) was performed to evaluate the expression of basic fibroblast growth factor (bFGF) and CD31. Anti-bFGF (cat. no. ZA-0568) and anti-CD31 (cat. no. ZA-0558) were purchased from Cell Signaling Technology (Boston, MA, United States) and OriGene Technologies Inc. (Rockville, MD, United States), respectively. IHC was performed as previously described (Tan et al., 2019). PBS was used for the negative controls. Staining was observed under a Nikon microscope (Nikon Corporation).

### Statistical Analysis

Data are presented as mean ± standard deviation ($\bar{x}+s$). The significance of differences between sample means was determined by least significant difference *t*-tests or *χ*^2^ test using SPSS19.0 software (SPSS Inc., Chicago, IL, United States).

## Results

### Photostability of PPIX-MED and Singlet Oxygen Yield

Photostability is a vital property of a photosensitizer and is determined by assessing photobleaching. Exposure of porphyrin derivatives to light may cause photochemical destruction of the tetrapyrrolic macrocycle, resulting in a decrease in the intensity of the Soret absorbance band at approximately 420 nm.

**FIGURE 3:**
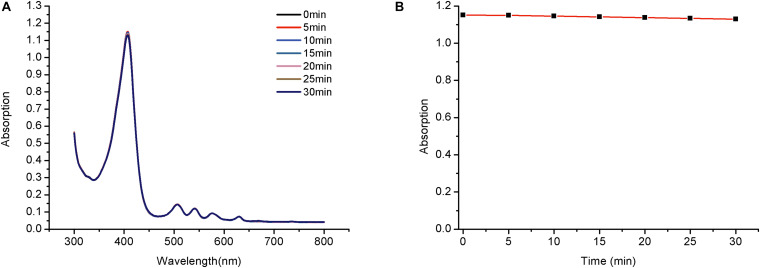
Photobleaching of PPIX-MED. Photostability of PPIX-MED with irradiation time (2 × 10^– 5^ mol/l PPIX-MED, 0.2 J/cm^2^ per min). **(A)** The absorption spectrum of compound PPIX-MED from 0 to 30 min. **(B)** The absorbance change of compound PPIX-MED against irradiation time at the maximum absorption wavelength.

The maximum absorption of PPIX-MED (at 418 nm) decreased slightly over time, but after irradiation with 650-nm light (6 J/cm^2^) for 30 min, the optical density of PPIX-MED changed by <1.9% (Figure 3). Thus, PPIX-MED is a photosensitizer with relatively high photostability.

An ideal photosensitizer should achieve a high singlet oxygen yield. In this study, DPBF was used as a reducing agent to trap singlet oxygen (^1^O_2_), and TPP was used as a reference compound to generate ^1^O_2_. [Φ_Δ_] for PPIX-MED reached 0.69 when a mixture of DPBF (2 × 10^–5^ mol/l) and PPIX-MED was irradiated with a 650-nm semiconductor laser (6 J/cm^2^). The DPBF decay rate was higher in the presence of PPIX-MED than TPP, revealing that PPIX-MED was more efficient than TPP in producing singlet oxygen.

### Uptake of PPIX-MED by Bacteria

**FIGURE 4:**
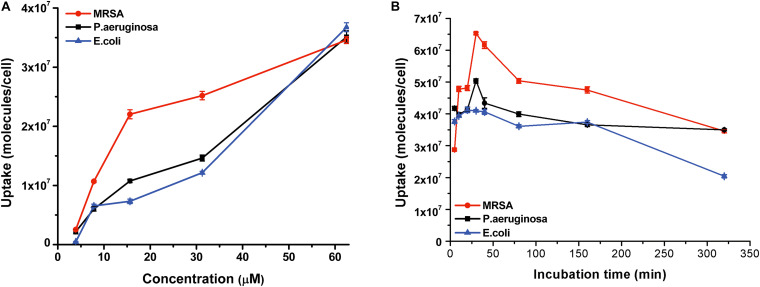
PPIX-MED was taken up by three bacterial strains. **(A)** Bacterial culture was incubated with PPIX-MED (3.91–62.5 μM) in the dark for 30 min at ambient temperature. **(B)** Bacterial culture was incubated with PPIX-MED (6.25 μM) for 320 min.

The aPDT efficacy shows a high correlation with the amount of the photosensitizer taken up by bacteria; accordingly, a long-enough incubation time is required (Xuan et al., 2019). Bacterial culture was incubated with PPIX-MED (6.25 μM) for 320 min, and the absorption of the photosensitizer by the bacteria was experimentally determined by spectrophotometry (Zhao et al., 2014). The uptake of PPIX-MED by the three bacterial species used in this study was dose- and time-dependent (Figure 4). Figure 4B shows that the three bacterial strains could absorb the maximum amount of the PPIX-MED in 30 min. Accordingly, 30 min was used as the incubation time for photoreaction and dark control reactions in subsequent *in vitro* and *in vivo* experiments in this work.

### MIC and MBC Determinations

The MIC and the MBC of PPIX-MED toward the test bacteria were studied. Bacterial suspensions (10^6^ CFU/ml) were incubated with PPIX-MED in the dark for 30 min at 37°C and then exposed to light (650 nm, 6 J/cm^2^). The concentration of PPIX-MED required to make the suspensions change visibly from turbid to clear was regarded as the MIC, while the concentration at which five colonies or less were observed on plates was regarded as the MBC. As shown in Table 1, PPIX-MED had high bacterial photoinactivation ability, with an MIC of 15.6 μM for each of MRSA, *E. coli*, and *P. aeruginosa*. The MBC values were 31.25 μM for MRSA, 31.25 μM for *E. coli*, and 62.5 μM for *P. aeruginosa*. Meanwhile, the dark toxicity of PPIX-MED was relatively low, with MIC and MBC values > 500 μM for the three strains. From the MBC values, it can be concluded that PPIX-MED–aPDT is very effective against *E. coli*, *P. aeruginosa*, and MRSA. Thus, PPIX-MED is a good compound to evaluate in the treatment of bacterially infected burn wounds.

**TABLE 1:** Minimum inhibitory concentration (MIC, μM) and minimum bactericidal concentration (MBC, μM) of PPIX-MED against methicillin-resistant *Staphylococcus aureus*, *Pseudomonas aeruginosa*, and *Escherichia coli*.

PPIX-MED	6 J/cm^2^		0 J/cm^2^	
	MIC	MBC	MIC	MBC
MRSA	15.6	31.25	500	>500
*P. aeruginosa*	15.6	62.5	>500	>500
*E. coli*	15.6	31.25	>500	>500

### Fractional Inhibitory Concentration Index

The Etest results on the effect of combining ceftriaxone sodium with PPIX-MED–aPDT are summarized in Table 2. The average FICI for MRSA for ceftriaxone sodium in combination with PPIX-MED was 1.0. Thus, no synergy or antagonism was seen with the combination. The average FICI for *P. aeruginosa* was 0.625, indicating that an additive effect occurred with the combination, and the average FICI for *E. coli* was 0.75, again indicating an additive effect.

**TABLE 2:** Etest minimum inhibitory concentrations (median and range) of ceftriaxone sodium and PPIX-MED alone and in combination.

	MIC (μg/ml)				FICI^▲^	Interpretation
	MIC_A_	MIC_A__(__with B__)_	MIC_B_	MIC_B__(__with A__)_		
MRSA	128 μg/ml	64 μg/ml	16 μM	8 μM	1	Indifference
*P. aeruginosa*	32 μg/ml	16 μg/ml	32 μM	4 μM	0.625	Indifference
*E. coli*	64 μg/ml	16 μg/ml	32 μM	16 μM	0.75	Indifference

*MIC_A_, ceftriaxone sodium; MIC_B_, PPIX-MED; MIC_A__(__with B__)_, ceftriaxone sodium with PPIX-MED; MIC_B__(__with A__)_, PPIX-MED with ceftriaxone sodium. ^▲^ The fractional inhibitory concentration index (FICI) data were interpreted using the following criteria: synergistic effect, FICI ≤ 0.5; additive effect, FICI 0.5–1.0; irrelevant effect, FICI 1.0–2.0; and antagonistic effect, FICI ≥ 2.0.*

### *In vitro* Photoinactivation of Bacteria Mediated by PPIX-MED

**FIGURE 5:**
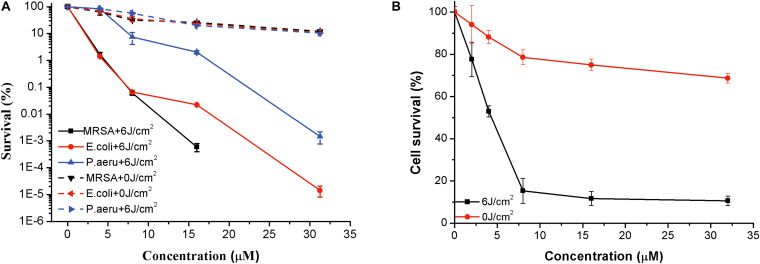
Phototoxicity and dark toxicity of PPIX-MED against **(A)** three bacterial strains and **(B)** NIH 3T3 (mouse fibroblast) cells. Each bacterial species and the fibroblasts were respectively incubated with PPIX-MED (0–31.25 μM) for 30 min, followed by exposure to 650-nm laser light (6 J/cm^2^) or darkness. The results are expressed as mean and standard deviation.

To delve into the photoinactivation effects of PPIX-MED against MRSA, *E. coli*, and *P. aeruginosa*, two experiments were performed: PPIX-MED treatment only and PPIX-MED + light treatment. Figure 5A shows the photodynamic efficacy of the photosensitizer against the three bacterial suspensions. In the PPIX-MED-only group, the bacterial survival rate decreased slightly, indicating that PPIX-MED alone had only a weak effect on the growth of the three bacterial strains tested in this study. When the bacterial suspensions were respectively incubated with PPIX-MED in the dark for 30 min at 37°C and then illuminated by the 650-nm laser (6 J/cm^2^), the photoinactivation effect of PPIX-MED against MRSA, *E. coli*, and *P. aeruginosa* was PPIX-MED dose dependent. A sharp decrease in bacterial survival fraction was observed with an increase of the PPIX-MED concentration. The activity of the bacteria was largely inhibited in the concentration range 0–31.25 μM PPIX-MED, and the inhibition rate was >99% at 31.25 μM for *E. coli* and *P. aeruginosa* (which are Gram-negative bacteria) and 15.6 μM for MRSA (a Gram-positive bacterium). The results reveal that PPIX-MED displayed highly efficient photoinactivation toward these three bacterial species.

### Phototoxicity of PPIX-MED Toward NIH 3T3 Cells

NIH 3T3 fibroblast cells were used as an example of normal mammalian cells to assess the phototoxicity of PPIX-MED via the MTT assay. NIH 3T3 cells were incubated with PPIX-MED in identical conditions to those used for MRSA, *E. coli*, and *P. aeruginosa*. The phototoxicity of PPIX-MED was concentration dependent (Figure 5B). In dark conditions, the cell survival of NIH 3T3 could reach >70% in the PPIX-MED concentration range 0–31.25 μM. After irradiation (6 J/cm^2^), the NIH 3T3 cell activity exceeded 10.6% at 31.25 μM PPIX-MED, at which concentration the three bacterial strains were almost fully eliminated.

### Confocal Laser Scanning Microscopy Images of Three Kinds of Bacteria Treated With PPIX-MED

**FIGURE 6:**
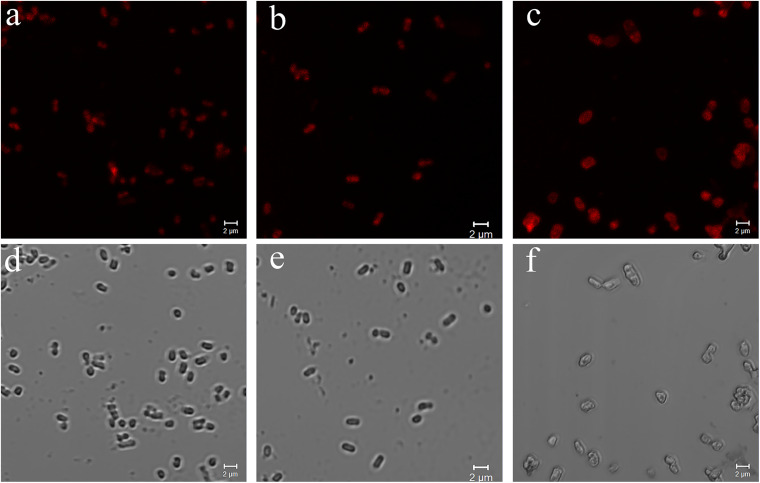
Confocal laser scanning microscopy images of **(a)** *Pseudomonas aeruginosa*, **(b)** *Escherichia coli*, and **(c)** methicillin-resistant *Staphylococcus aureus* (MRSA) and optical images of **(d)** *P. aeruginosa*, **(e)** *E. coli*, and **(f)** MRSA. The strains were imaged after incubation for 30 min with PPIX-MED (15.6 μM) in phosphate-buffered saline. Excitation was at 405 nm. Scale bars = 2 μm.

Uptake of PPIX-MED by the three species of bacteria was examined by confocal laser scanning microscopy. Porphyrin conjugates emit red fluorescence at 650 nm when excited at 405 nm, which can be readily monitored with a fluorescence microscope system (Hamblin et al., 2003). The images in Figure 6 confirm that PPIX-MED was internalized by the bacteria after incubation for only 30 min. The fluorescence imaging of *E. coli*, *P. aeruginosa*, or MRSA incubated with PPIX-MED was consistent with the dose-dependent photoinactivation effects.

### *In vivo* Experiments

#### The Wounds and Healing Rates

To monitor the wound healing process, the leakage quantity of burn wounds, the presence of secretions, and healing range were observed at different times after infection. At 1 day after infection, the wounds of rats in the treated groups and the control group (no treatment) showed pale necrosis, the skin became hard and convex, and the wound surface showed bloody scabs and necrotic tissue coverage and congestion. At 4 days after infection, the untreated (control group) animals showed wound decay and ulceration. However, in the aPDT group and the aPDT-combined-with-antibiotic group, the skin was smooth, the wound scab was black and dry, and the wounds were better than in the antibiotic-only treatment group. At 10 days after infection, the wounds of the rats in the untreated control group were beginning to heal, but they were still very large and there was pus under the scab. Wound healing in the antibiotic-only treatment group was better than that in the untreated group. The scab did not fall off. It was gray, and there was pus underneath. In the aPDT and aPDT-combined-with-antibiotic treatment groups, all the scabs fell off, and new pink tissues were exposed.

**FIGURE 7:**
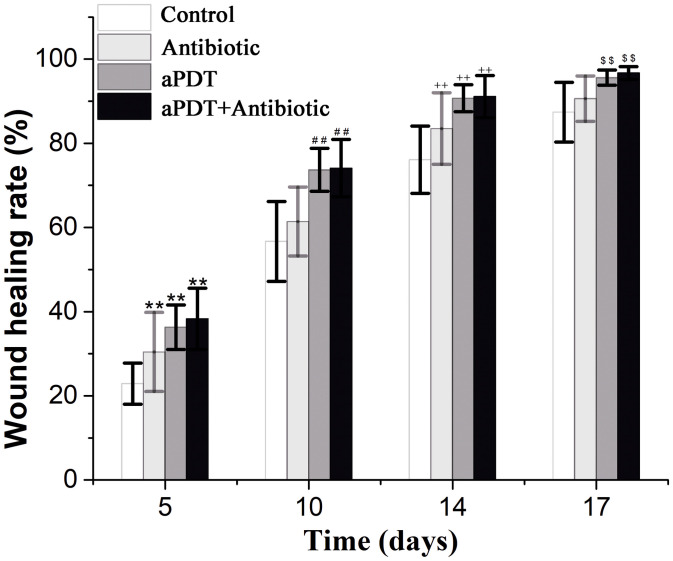
Wound healing rate of each group on the 5th, 10th, 14th, and 17th day post-infection [*p < 0.05, **p < 0.01 vs. control group (5 days); ^#^*p* < 0.05, ^##^*p* < 0.01 vs. control group (10 days); ^+^*p* < 0.05, ^++^*p* < 0.01 vs. control group (14 days); ^$^*p* < 0.05, ^$$^*p* < 0.01 vs. control group (17 days)].

For *in vivo* aPDT experiments, about five or 10 times the MIC is generally chosen as the working dose (Xu et al., 2016). On the basis of preliminary experiments, 187.5 mM of PPIX-MED, four times the MBC against *P. aeruginosa*, was chosen as the working dose for this study. The antibacterial effect of PPIX-MED–aPDT *in vivo* was analyzed. As shown in Figure 7, on days 5, 10, 14, and 17 post-infection, the wound healing rate of the treatment groups was higher than that of the untreated control group (*p* < 0.01). The rats in the aPDT + antibiotic therapy group exhibited faster wound healing than those in the antibiotic-therapy-only group (*p* < 0.01) or the aPDT treatment group. On day 17 post-infection, the wounds in the aPDT + antibiotics group had healed (96.7 ± 1.5); the healing rate was significantly better than that in the antibiotic-only treatment group (*p* < 0.01) and the aPDT treatment group, respectively. The healing rate of the aPDT treatment group was superior to that of the antibiotic-only treatment group (*p* < 0.01). Therefore, aPDT combined with antibiotics was most efficient in the treatment of mixed bacterial infection of burn wounds, and aPDT treatment was also somewhat effective.

#### Viable Bacteria in Wound Tissue

**FIGURE 8:**
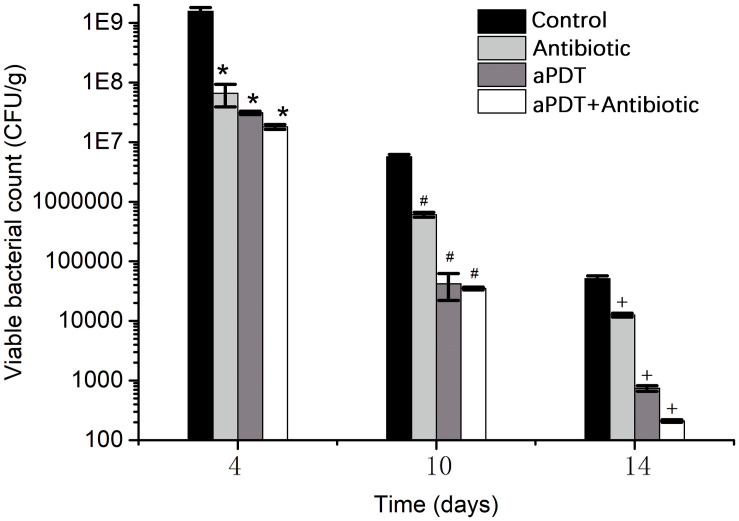
The viability of bacteria in burn wound tissue of rats in each group on the 4th, 10th, and 14th day post-infection [*p < 0.05 vs. control group (4 days); ^#^*p* < 0.05 vs. control group (10 days); ^+^*p* < 0.05 vs. control group (14 days)].

The viability of bacteria in wound tissue was determined as an index of the bactericidal effect of PPIX-MED–aPDT treatment (Figure 8). The control group (i.e., wounds without any treatment) exhibited greater viability of MRSA, *P. aeruginosa*, and *E. coli* at each observation time point; on days 4, 10, and 14 post-infection, all the treatment groups exhibited an obvious reduction in bacterial viability compared with the controls (*p* < 0.05). In the treatment groups, bacterial viability decreased in the order aPDT + ceftriaxone group > aPDT group > ceftriaxone group. This suggests that PPIX-MED–aPDT has a bactericidal effect against MRSA, *P. aeruginosa*, and *E. coli* in burn wounds, and aPDT combined with antibiotics is more efficient in the treatment of burn infection.

#### Bacteria in Blood Culture and Animal Survival

As shown in Table 3, at 4 days after infection, the positive detection rate of bacteria in blood culture analysis for the aPDT + ceftriaxone (70%) and aPDT (88.9%) groups was lower than that in the ceftriaxone (100%) and control groups (100%). At 10 days, the aPDT + ceftriaxone (0%), aPDT (33.3%), and ceftriaxone groups (71.4%) showed a reduction of positive blood cultures compared with the control group (100%; *p* = 0.004). At 14 days, the rate of bacteria-positive blood cultures was still zero for the aPDT + ceftriaxone group and was 11.1% for the aPDT group and 42.8% for the ceftriaxone group; however, the rate in the control group remained high (80%).

**TABLE 3:** Rate of detection of bacteria in blood cultures at 4, 10, and 14 days after infection ($\bar{x}+s$).

Groups	4 days	10 days	14 days
Control	100.0	100.0^a^	80.0^a^
Antibiotic	100.0	71.4^a▲^	42.8^a▲^
aPDT	88.9	33.3^b▲^	11.1^b▲^
aPDT + Antibiotic	70.0	0.0^b^	0.0^b^

*At the same time, the difference in the α = 0.05 level between the different groups in the upper-right corner of the digital right angle was statistically significant; ^▲^ showing no difference between statistical significance (ignore the letters that are different).*

**FIGURE 9:**
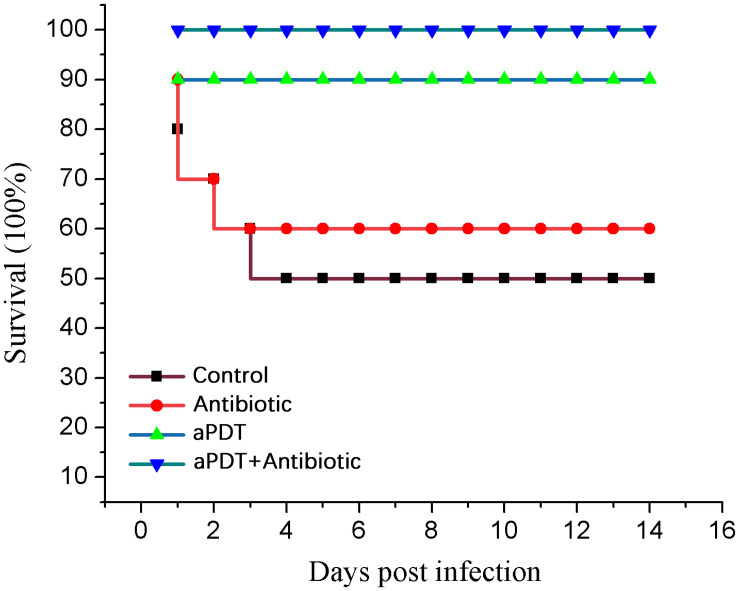
Survival of treated and untreated rats.

In the control group, five rats died on days 1–4 post-infection. In the antibiotic treatment group, four rats died on days 1–3. In the aPDT treatment group, one rat died (on day 1). All rats in the aPDT + ceftriaxone group survived the experiment. These data indicate that PPIX-MED–aPDT was the most efficient approach to the treatment of mixed bacterial infection in a burn wound (Figure 9).

### ELISA

**FIGURE 10:**
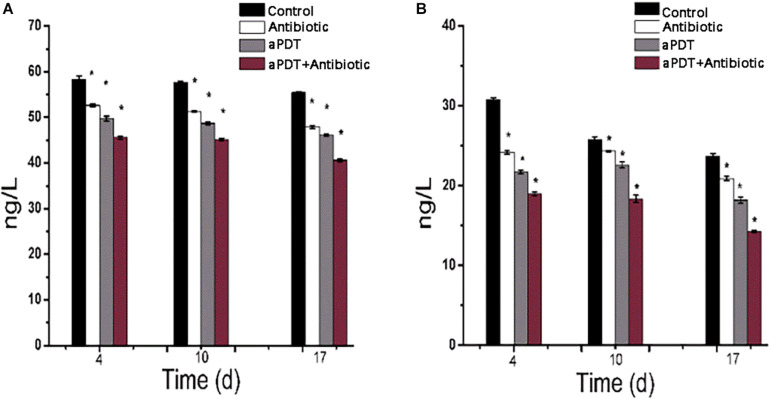
Expression of TNF-α and IL-6 as determined by ELISA in burn wound tissue of rats. **(A)** TNF-α levels at 4, 10, and 17 days after infection. **(B)** IL-6 levels at 4, 10, and 17 days after infection.

ELISA was used to investigate tissue TNF-α and IL-6 concentrations in skin tissue after treatment (Figure 10). The TNF-α and IL-6 levels gradually decreased with time post-infection. The aPDT + antibiotics-treated group exhibited an obvious reduction in TNF-α and IL-6 levels (*p* < 0.01) compared with the no-treatment (control) group and the other treatment groups; at a given time point, the level of TNF-α and IL-6 in the groups was control > antibiotic > aPDT > aPDT + antibiotics. These results suggest that aPDT, especially when combined with antibiotic therapy, can inhibit the expression of IL-6 and TNF-α in burn tissues, which will reduce the inflammatory reaction in the tissues and help with wound healing.

### Expression of bFGF and CD31 in Burn Wound Tissue

**FIGURE 11:**
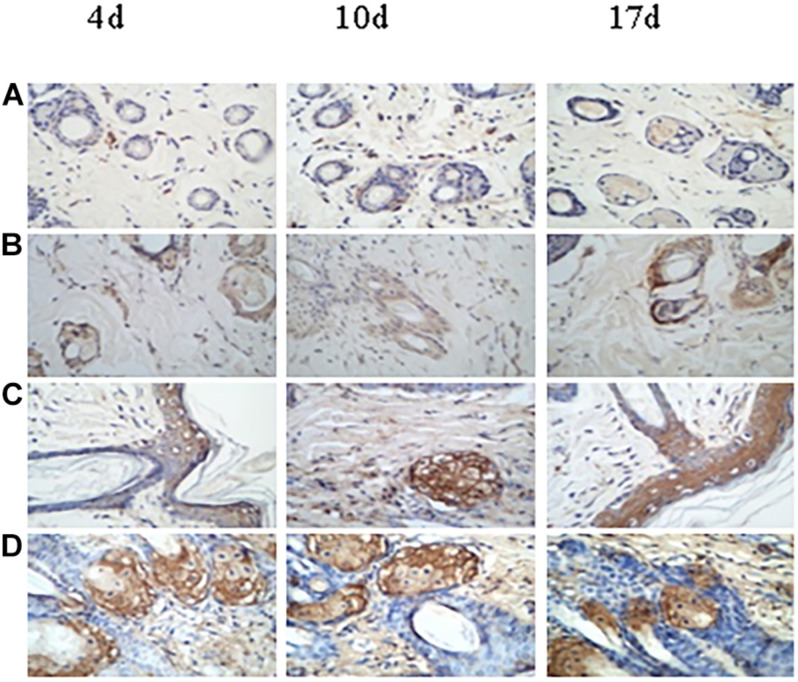
Expression of bFGF in burn wound tissue by time (days post-infection) in treated and untreated rats. **(A)** Control group. **(B)** Antibiotic treatment group. **(C)** aPDT treatment group. **(D)** aPDT + antibiotic treatment group.

Table 4 and Figure 11 show the expression of bFGF at 4, 10, and 17 days post-infection in the various groups. At 4 days, the expression of bFGF in the antibiotic-only (36.33 ± 5.71), aPDT-only (50.50 ± 14.22), and aPDT + antibiotics (75.83 ± 23.25) treatment groups was higher than that in the control group (11.67 ± 2.07) (*p* < 0.05). At 10 days, the values were 41.33 ± 3.50, 91.00 ± 7.27, and 99.67 ± 9.27, respectively, compared with 16.67 ± 2.66 for the control group (*p* < 0.05). At day 17, the expression of bFGF in the antibiotic-only (48.33 ± 17.95), aPDT-only (51.83 ± 14.95), and aPDT + antibiotics (43.67 ± 4.97) groups remained higher than that in the control group (39.00 ± 5.90).

**TABLE 4:** Expression of bFGF in tissues of burn wound sites as determined by immunochemistry.

Group	4 d	10 d	17 d
Control	11.67 ± 2.07^a^	16.67 ± 2.66^a^	39.00 ± 5.90
Antibiotic	36.33 ± 5.71^b^	41.33 ± 3.50^b^	48.33 ± 17.95
aPDT	50.50 ± 14.22^b^	91.00 ± 7.27^c^	51.83 ± 14.95
aPDT + Antibiotic	75.83 ± 23.25^c^	99.67 ± 9.27^c^	43.67 ± 4.97

*The letter is completely different between p < 0.05.*

At 4 and 10 days post-infection, the bFGF expression level in the aPDT + antibiotic group was obviously higher than that in the antibiotic and the aPDT treatment groups (*p* < 0.01). The expression of bFGF in the aPDT treatment group was higher than that in the antibiotic treatment group (4 days, *p* > 0.05; 10 days, *p* < 0.01). The expression level in the control and the antibiotic groups increased throughout the healing process. However, in the aPDT treatment group and the aPDT + antibiotic group, bFGF expression first increased and then decreased, being highest at 10 days post-infection among the timepoints that we measured: 99.7 ± 9.3 in the aPDT + antibiotic treatment group and 91.0 ± 7.3 in the aPDT group.

**FIGURE 12:**
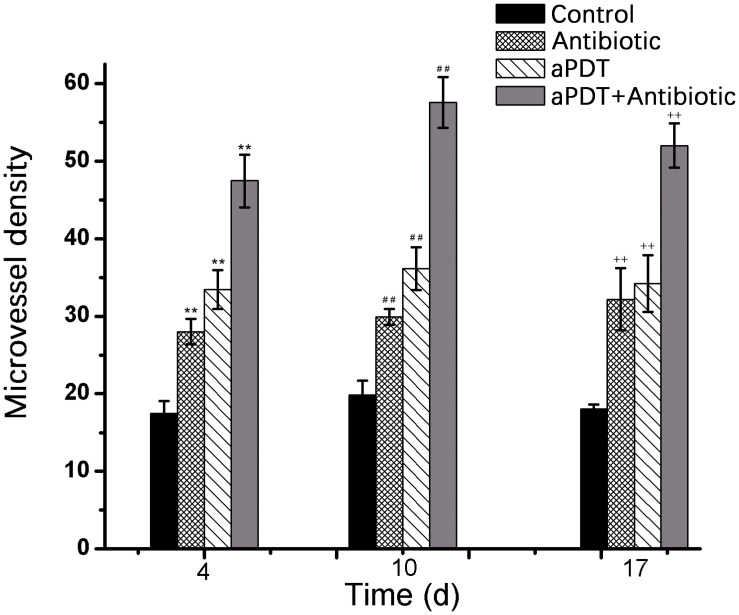
Blood vessel density (measured by the expression of CD31) in tissues of burn wound sites as determined by immunochemistry [*p < 0.05, **p < 0.01 vs. control group (4 days); ^#^*p* < 0.05, ^##^*p* < 0.01 vs. control group (10 days); ^+^*p* < 0.05, ^++^*p* < 0.01 vs. control group (17 days)].

On days 4, 10, and 17 post-infection, the new blood vessel density (MVD), determined by the expression of CD31, was significantly higher in the treatment groups than in the control group (*p* < 0.01) (Figure 12). MVD in the aPDT + antibiotics group was significantly higher than that in the aPDT-only treatment group and the antibiotic-only group (*p* < 0.01). The MVD in the aPDT-only treatment group was significantly higher than that in the antibiotics-only group (*p* < 0.01 at 4 and 10 days post-infection). The new MVD in the aPDT + antibiotic group reached its observed maximum on day 10.

### Evaluation of Side Effects of Using aPDT + Antibiotics

We examined the potential *in vivo* toxicity of PPIX-MED–aPDT and aPDT + antibiotics treatments using the organ index in rats (Table 5). We harvested major organs: heart, liver, spleen, lung, kidney, and thymus. Compared with normal rats, we did not detect any overt signs of toxic side effects or changes in body weight or organ weight with the PPIX-MED–aPDT treatment, suggesting that the PPIX-MED–aPDT treatment was relatively safe to administer. However, the liver weight of the rats in the antibiotic treatment group and the aPDT + antibiotics group was lower than that in the other groups (*p* < 0.01), which may be caused by the toxicity of ceftriaxone sodium to the liver.

**TABLE 5:** The organ index in rats after treatment.

Group	Weight (g)	Heart (mg/g)	Liver (mg/g)	Spleen (mg/g)	Lung (mg/g)	Kidney (mg/g)	Thymus index (mg/g)
Normal	286 ± 42	0.40 ± 0.02	4.05 ± 0.40	0.38 ± 0.10	0.70 ± 0.10	0.87 ± 0.10	0.18 ± 0.02
Control	263 ± 22	0.40 ± 0.10	4.60 ± 0.50	0.57 ± 0.10	0.73 ± 0.15	0.94 ± 0.20	0.16 ± 0.05
Antibiotic	283 ± 22	0.36 ± 0.05	3.50 ± 0.40^▲^	0.40 ± 0.20	0.70 ± 0.03	0.80 ± 0.04	0.17 ± 0.04
aPDT	292 ± 22	0.38 ± 0.03	4.20 ± 0.30	0.38 ± 0.07	0.60 ± 0.04	0.80 ± 0.05	0.17 ± 0.04
aPDT + Antibiotic	281 ± 16	0.38 ± 0.05	3.80 ± 0.40^▲^	0.42 ± 0.07	0.70 ± 0.04	0.86 ± 0.05	0.17 ± 0.03

*Compared with control, ^▲^ means p < 0.05.*

In control group rats, the body weight was lower than it was in the treatment groups. When dissecting the rats, many infections were observed on the spleen, and the small intestine adhered to the abdominal wall on both sides. Rats in the antibiotic-only treatment group also showed symptoms such as a grossly enlarged spleen and intestinal adhesions. These symptoms indicated that the experimental animals had different degrees of bacterial organ infections.

## Discussion

Complications caused by drug-resistant bacterial infection after burns are a major factor in the death of patients. Bacterial attachment to an open wound during the healing process is a prominent etiological factor of sepsis and multiple organ dysfunction syndrome (MODS), for example (Garcia et al., 2010). As such, anti-infection treatment is important for burn patients. Furthermore, the emergence of high antimicrobial resistance among bacterial pathogens has made the management of treatment of postoperative wound infections difficult (Andhoga et al., 2002; Nanda and Saravanan, 2009). Healing of burn wounds is a complex process that involves the response of several local and systemic tissues and is regulated by many different cellular and humoral factors. It normally proceeds in four overlapping phases: inflammation, granulation, matrix formation, and remodeling (Garcia et al., 2010).

*In vitro* (Thakuri et al., 2011; ElZorkany et al., 2019; Choi et al., 2020) and *in vivo* (de Vasconcelos Catão et al., 2015; Mai et al., 2017) studies have demonstrated that aPDT has anti-infection properties and effects on wound healing. A meta-analysis of aPDT indicated that it is highly effective in promoting tissue repair (Garcia et al., 2010; Mai et al., 2017). There are several reports in the literature on the beneficial effects of aPDT on local vascularization, edema, pain, and inflammation as well as the deposition and organization of both extracellular matrix and collagen (Garcia et al., 2010), but there are few published reports (Boluki et al., 2017) on the effect of aPDT-combined-with-antibiotic treatment on the healing of burns.

In our study, both aPDT alone and aPDT-combined-with-antibiotic treatment were used. We used a rat model with severe burns, infected by a mixture of pathogenic bacteria. Wounds treated with aPDT or aPDT combined with antibiotics healed in 5–17 days, faster than wounds in the control (untreated) group or the group treated with antibiotic alone. Moreover, half the rats in the control (untreated) group died within 4 days of infection, and 40% of the rats in the antibiotic-only treatment group died early in the experiment (days 1–3), but no rats in the aPDT + antibiotics treatment group died, and only one died in the aPDT-only group (on the first day after burning and infection). Immunohistochemical studies showed that aPDT and aPDT combined with antibiotic promoted a high expression of bFGF before day 10; on day 10, the expression of bFGF was lower, whereas the expression level in the control group and the antibiotic-only group continued to increase at that timepoint. This may suggest that aPDT and aPDT combined with antibiotic can promote fibroblast proliferation that enhances wound healing (Garcia et al., 2010).

The viable bacteria in wound tissue and in blood were determined as an index of the bactericidal effects of aPDT and aPDT combined with antibiotics. On the 4th, 10th, and 14th day after infection, the aPDT-combined-with-antibiotics treatment group exhibited an obvious reduction in bacteria compared with the control group and the aPDT-only treatment group and the antibiotic-only group. This experiment confirmed that aPDT can significantly reduce the number of bacterial colonies under phase callus, increasing the rate of wound healing, and promote healing efficacy.

TNF-α is a crucial contributing factor to inflammation-mediated pathophysiology (Brüünsgaard and Pedersen, 2003; Ashcroft et al., 2012). The pleiotropic cytokine IL-6 is produced by macrophages, dendritic cells, mast cells, and other innate immune cells. TNF-α and IL-6 have long been considered as markers of inflammation (Nissen et al., 1996). In the present study, aPDT and aPDT-combined-with-antibiotic treatment decreased inflammation factor secretion; that is, it significantly reduced the scalded tissue concentration of TNF-α and IL-6. Neovascularization is an important part of granulation, providing nutrition and transporting metabolites to a wounded area, which plays an important role in the process of wound healing. Angiogenesis is critically important for the delivery of nutrients, oxygen, and inflammatory cells to the area of wound, which therefore contribute to promote wound healing (Salo et al., 2007). CD31 is a sensitive marker for the detection of neovascularization (Musumeci et al., 2015). The results here showed a higher expression of CD31 in the aPDT-combined-with-antibiotics group compared with the aPDT-only treatment group and the antibiotic-only group (*p* < 0.01). In the aPDT-combined-with-antibiotics group, the new blood vessel density reached its observed maximum on day 10 and was lower on day 14. The results showed that aPDT-combined-with-antibiotic treatment can promote neovascularization in burn wounds. Salo et al. (2007) showed that wound neovascularization is the main determinant of burn wound healing.

The major advantages of aPDT are its specific effect on target cells, a lack of collateral effects, activity only on exposure to light, and inability of bacteria to develop resistance to this type of killing (Zolfaghari et al., 2009; Sperandio et al., 2013). Further studies should be carried out to better understand the action of aPDT-combined-with-antibiotics in the healing process of third-degree burns because there is little literature on this subject. Moreover, it should be noted that the present safety evaluation of PPIX-MED is somewhat limited, and this should be expanded in a series of rigorous assessments before PPIX-MED is used clinically.

## Conclusion

aPDT and aPDT-combined-with-antibiotic treatment showed beneficial effects in accelerating the healing process of bacterially infected third-degree burns of rats. These treatments stimulate macrophages to release chemical mediators, cytokines, and growth factors, which, in turn, stimulate and increase the production of connective tissue and create a new supply of blood vessels to nourish the wound site and promote remodeling. There was a histological tendency for better cicatrization after the use of aPDT combined with antibiotics in burn healing. The synergistic effect of aPDT-combined-with-antibiotic treatment could be promising for the management of third-degree burn skin infections.

## Data Availability Statement

The original contributions presented in the study are included in the article/supplementary material, further inquiries can be directed to the corresponding author/s.

## Ethics Statement

The animal study was reviewed and approved by the Laboratory Animal Management Committee/Laboratory Animal Welfare Ethics Committee, Institute of Radiation Medicine, Chinese Academy of Medical Sciences.

## Author Contributions

TL and GH performed conceptualization. ZZ, TL, and GH performed data curation. ZZ and GH performed formal analysis. GH performed funding acquisition, project administration, and supervision. JZ, ZZ, and GH performed investigation. LZ, TL, and GH performed methodology. ZZ, TL, and GH performed resources. ZX, YW, JM, and GH performed validation. ZZ and GH performed visualization and writing – original draft. ZZ, TL, and GH performed writing – review and editing. All authors contributed to the article and approved the submitted version.

## Conflict of Interest

The authors declare that the research was conducted in the absence of any commercial or financial relationships that could be construed as a potential conflict of interest.
